# *Eucommia ulmoides* extract regulates oxidative stress to maintain calcium homeostasis and improve diabetic osteoporosis

**DOI:** 10.1002/fsn3.4413

**Published:** 2024-08-20

**Authors:** Jie Shen, Yichen Gao, Yuyao Deng, Zhaoxin Xia, Xia Wang, Xianyi He, Yun He, Binbin Yang

**Affiliations:** ^1^ Department of Oral and Maxillofacial Surgery, The Affiliated Stomatological Hospital Southwest Medical University Luzhou Sichuan China; ^2^ Oral and Maxillofacial Reconstruction and Regeneration of Luzhou Key Laboratory Luzhou Sichuan China; ^3^ National Engineering Research Center for Biomaterials Sichuan University Chengdu China

**Keywords:** bone metabolism, calcium homeostasis, diabetic osteoporosis, *Eucommia ulmoides* aqueous extract, oxidative stress

## Abstract

Diabetic osteoporosis (DOP) is a secondary disease that severely affects the health and quality of life of patients with diabetes mellitus. This study aimed to explore the bone protective effect of aqueous extract of *Eucommia ulmoides* (EUL) in DOP mice. DOP mice were established using a high‐sugar, high‐fat diet and streptozotocin (STZ) (35 mg/kg for three consecutive days), and the EUL aqueous extract (2.5 g/kg/day) was orally administered for 6 weeks. The serum levels of oxidative stress‐related factors, calcium, and phosphorus were assessed using biochemical assays. The osteoprotective effect of EUL was assessed using micro‐computer tomography, three‐point bending assay, histological analysis, and immunoblotting. Quantitative real‐time polymerase chain reaction and western blotting were performed to detect the expression levels of calcium transport channel factors in the kidney and small intestine tissues. Furthermore, the expression levels of nuclear factor erythroid 2‐related factor 2 (*Nrf2*) and heme oxygenase‐1 (*HO‐1*) in the femur, kidney, and small intestine tissues were detected using western blotting and quantitative real‐time polymerase chain reaction. EUL aqueous extract reduced blood glucose levels, increased body weight, and relieved symptoms in DOP mice (*p* < .05). It also increased bone mineral density, improved the bone microstructure, decreased the number of femoral osteoclasts, and increased the expression of femoral Runx2 and Bmp2 in DOP mice (*p* < .01). After 6 weeks of EUL aqueous extract administration, serum levels of SOD, CTA, calcium, and phosphorus were upregulated, whereas MDA levels were decreased (*p* < .01). The aqueous EUL extract also upregulated the expression of TRPV5, PMCA‐1b, and CaBP‐9 k in the kidney and small intestine of DOP mice (*p* < .01). Furthermore, the expression of *Nrf2* and *HO‐1* in the kidney, small intestine, and femur tissues was increased (*p* < .01). EUL aqueous extract reduced blood glucose levels in DOP mice and regulated oxidative stress through the *Nrf2/HO‐1* pathway, thereby maintaining calcium homeostasis and ultimately improving bone quality. Our study suggested that EUL aqueous extract may be effective in the treatment of DOP.

## Graphical abstract

The schematic diagram illustrates the underlying mechanism of EUL aqueous extract in ameliorating bone mass in DOP mice. Administration of EUL aqueous extract in DOP mice eliminates oxidative stress in body by regulating the Nrf2/HO‐1 pathway. This promoted TRPV5/CaBP‐D9k/PMCA‐1b signaling in the intestine and kidney, which maintained calcium homeostasis in DOP mice and ultimately ameliorated DOP.

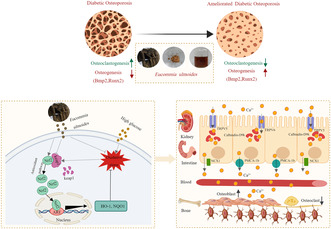

## 1 INTRODUCTION

Diabetes mellitus (DM) is characterized by hyperglycemia due to insulin dysfunction and is associated with many complications (Ma et al., 2020; Sumida et al., 2020). Numerous studies have confirmed that hyperglycemia is an important risk factor for fractures and osteoporosis (Shanbhogue et al., 2016; Zhao et al., 2021). Diabetic osteoporosis (DOP), a secondary disease of diabetes, is characterized by an increased risk of fractures, which severely affects the health and quality of life of patients with diabetes (Ma et al., 2016; Zhao et al., 2020). Sustained hyperglycemia caused by diabetes increases the activity of osteoclasts by increasing the expression of osteoclast receptor activator and reduces the differentiation ability of osteoblasts by reducing the proliferation and activity of osteoblasts, leading to bone loss (Rathinavelu et al., 2018). In addition, persistent hyperglycemia increases the decomposition ability of collagen fibers by destroying them in the bone tissue, resulting in a decrease in the distribution of bone trabeculae, which triggers the development of osteoporosis (Lee et al., 2021).

Sustained hyperglycemia can cause calcium metabolism disorders in the body, which impair the bone microstructure and hinder bone remodeling (Nie et al., 2020; Wongdee et al., 2017). Calcium homeostasis refers to a balanced state of calcium storage in the human body over a period of time, which makes a valuable contribution to the regulation of bone development and metabolism (Nie et al., 2020). It is maintained by the synergistic action of the calcium regulatory organs, including intestinal calcium absorption, renal calcium reabsorption, and bone metabolism (Matikainen et al., 2021; Weaver & Peacock, 2011, 2019). Sustained hyperglycemia in DM triggers an oxidative stress response that promotes pathological changes in calcium‐regulating organs, such as thinning and irregularity of bone trabeculae, reduction of mineralized nodules, increased bone resorption, and down‐regulation of renal and small intestinal calcium‐translocating channel expression, which affects the regulation of calcium homeostasis (Liu et al., 2018; Naidoo et al., 2022). Hyperglycemia‐triggered oxidative stress alters the expression of molecular proteins involved in the intracellular calcium pathway and inhibits the transport of calcium ions from the tubule lumen to the bloodstream, leading to decreased calcium absorption and increased calcium loss (Rivoira et al., 2015). In addition, oxidative stress triggered by hyperglycemia inhibits osteoblast differentiation and proliferation, promotes osteoclast differentiation, facilitates increased bone resorption, and ultimately leads to bone metabolic disorders (Zhu et al., 2022). Therefore, improving the structure and function of calcium‐regulating organs can help maintain the balance of calcium homeostasis, which ameliorates DOP.

*Eucommia ulmoides* (EUL), the daily diet of patients with osteoporosis, has been used for brewing with boiling water for more than 3000 years in Asia (Huang et al., 2021). Modern pharmacological research has revealed that EUL has significant effects on diabetes, osteoporosis, and other diseases (Hussain et al., 2016). Meanwhile, EUL contains various phytochemicals, including lignans, phenols, terpenes, and flavonoids, which have antioxidant, anti‐inflammatory, and anti‐apoptotic effects (Deyama et al., 2001). A recent study discovered that EUL alleviates diabetes‐induced renal injury by inhibiting the formation of advanced glycation end products (AGEs) and their cross‐linking with receptors of advanced glycation end products (RAGE) by activating the nuclear factor erythroid 2‐related factor 2 (*Nrf2*) signaling pathway, which reduces the oxidative stress response (Do et al., 2018). EUL flavones reduce oxidative stress by upregulating the expression of *Nrf2*/heme oxygenase‐1 (*HO‐1*) pathway, which alleviates paraquat‐induced intestinal injury (Xiao et al., 2019). Moreover, flavonoids extracted from EUL reduce oxidative stress by upregulating the expression of *Nrf2*/*HO‐1* pathway, which slows down bone loss (Xiao et al., 2023). Based on these findings, we hypothesized that the EUL aqueous extract could regulate oxidative stress through the *Nrf2*/*HO‐1* pathway and maintain calcium homeostasis in the kidney, intestine, and bone tissues, thereby ameliorating DOP.

## 2 METHODS AND MATERIALS

### 2.1 Animal model

**FIGURE 1:**
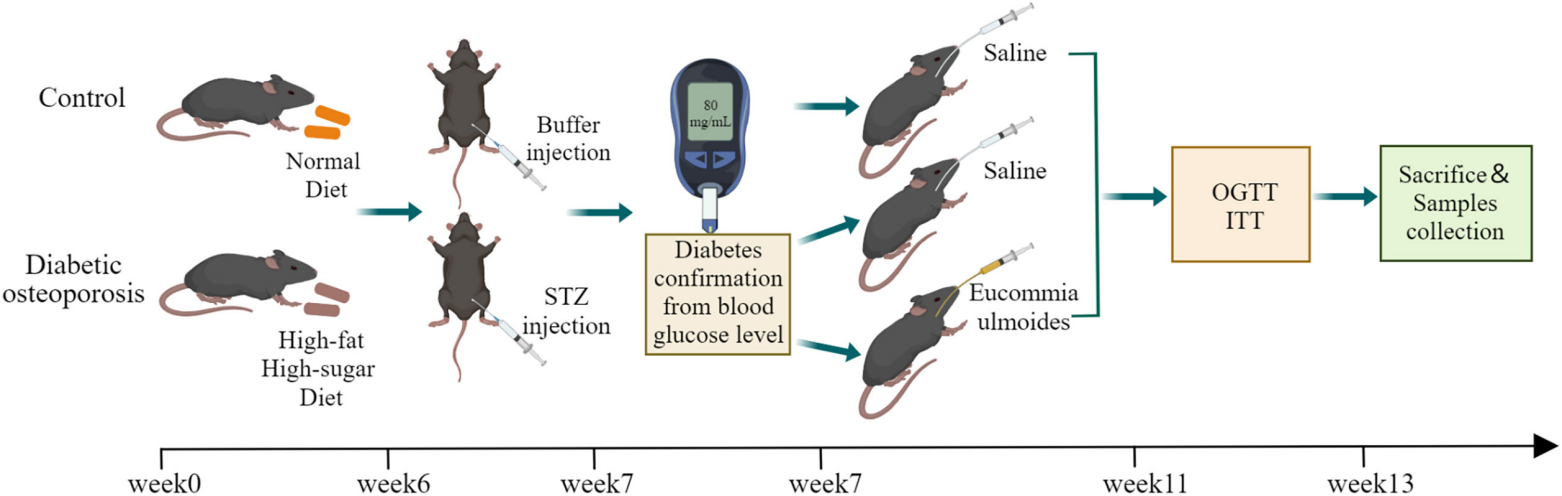
Schematic diagram of the research experiment design. Buffer, sodium citrate; ITT, insulin tolerance test; OGTT, oral glucose tolerance test; STZ, streptozotocin.

The animal study protocol was reviewed and approved by the Institutional Ethics Committee of the Affiliated Stomatological Hospital, Southwest Medical University (certificate number, 20221125–015). Six‐week‐old C57BL/6 male mice from the Experimental Animal Centre of Southwest Medical University were randomly divided into model and control groups. The model group was fed a high‐fat and high‐sugar diet for 2 months and then intraperitoneally injected with a small amount of streptozotocin (35 mg/kg) multiple times. The control group was fed a common diet for 2 months, and then intraperitoneally injected with the same dose of sodium citrate buffer. One week later, the mice with blood sugar levels higher than 16.7 mmol/L were randomly divided into the DOP and EUL groups. The EUL group was orally administered EUL aqueous extract (2.5 g/kg/day) daily (Xie et al., 2015), whereas the control and the DOP groups were orally administered equal amounts of distilled water for 6 weeks. Blood glucose levels and body weights of all mice were monitored weekly during the experiment (Figure 1).

### 2.2 Aqueous extract preparation of EUL

**FIGURE 2:**
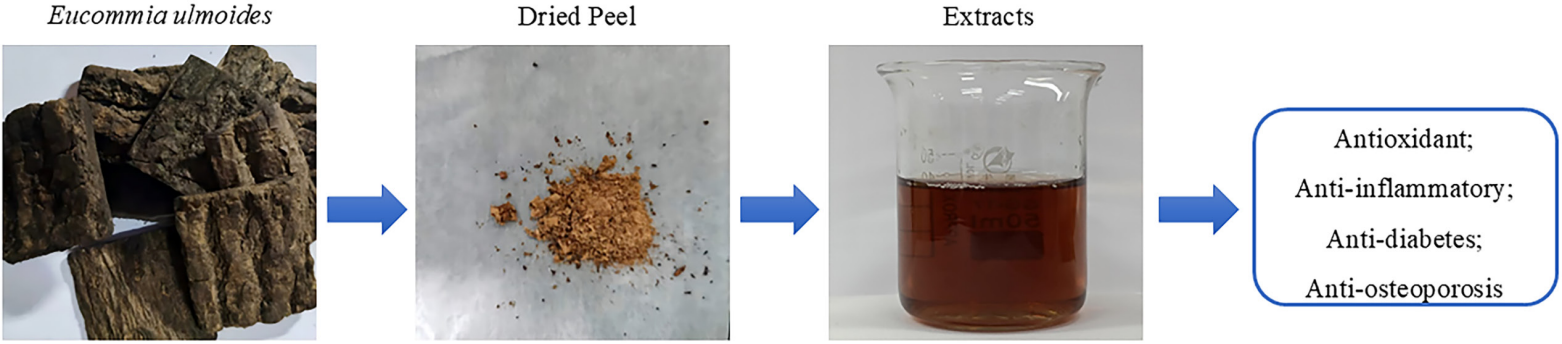
Preparation of EUL aqueous extract.

First, EUL bark (100 g) was soaked in distilled water (1 L) for 2 h and then boiled for 1 h. The solution was then collected, centrifuged at low temperature (5000× *g* for 15 min), continuously filtered, dried, and concentrated at constant temperature of 60°C, freeze‐dried, weighed, and finally stored at −80°C (Figure 2) (Zhao et al., 2020).

### 2.3 Oral glucose tolerance (OGTT) and insulin tolerance tests (ITT)

In the 11th week of the experiment, all mice were orally gavaged with glucose (1.0 g/kg) after being fasted overnight (12 h) or intraperitoneal injection of pancreatic insulin (0.75 U/kg) after 2 h of fasting. Blood was collected from the tail vein at regular intervals for the glucose content assay, and the area under the curve was calculated.

### 2.4 Specimen collection and preservation

After collecting urine, all mice were subjected to cardiac puncture and blood collection under gas anesthesia and centrifuged at a low temperature to obtain the serum. Subsequently, the heart, lungs, spleen, liver, pancreas, small intestine, bilateral kidneys, and femur of the mice were removed for further study, and the heart, lungs, spleen, liver, and bilateral kidneys were weighed to calculate heart (heart weight/body weight ratio), lungs (lungs weight/body weight ratio), spleen (spleen weight/body weight ratio), liver (liver weight/body weight ratio), and kidney indices (kidney weight/body weight ratio).

### 2.5 Determination of biochemical parameters

Serum Ca^2+^, P^2+^, SOD, MDA, CAT, urine Ca^2+^, and P^2+^, and bone Ca^2+^ and P^2+^ levels were determined using commercial kits (Nanjing Jiancheng Bioengineering Institute, Nanjing, China or Beyotime, Shanghai, China) according to the manufacturer's protocols.

### 2.6 Microcomputed tomography (micro‐CT) assessment

The mice femurs were fixed with 10% paraformaldehyde. The desktop‐type X‐ray micro‐CT imaging system nanoVoxel‐1000 (Sanying Precision Instruments Co. Ltd, Shenzhen, China) with a voltage of 110 kV, current of 500 μA, and an exposure time of 200 millisecond was used to measure the bone surface area/bone volume (BS/BV), bone volume/tissue volume (BV/TV), trabecular number (Tb. N), trabecular thickness (Tb. Th), and trabecular separation (Tb. Sp) of all femurs.

### 2.7 Biomechanical analysis

The biomechanical properties of the femur were evaluated using an electronic universal testing machine (RGWF4005; Shenzhen Reger Instrument Co., Ltd., China). The load on the femoral fracture was defined as the ultimate load. The elastic modulus and flexural strength of the femurs were also recorded.

### 2.8 Calcein double‐labeling

All mice were intraperitoneally injected with calcein (5 mg/kg) in the twelfth and second days before execution, bilateral femurs were isolated, and hard tissue sections were made. The distance between the two calcein concentrations in the metaphysis was observed using a fluorescence microscope (Olympus BX‐60; Tokyo, Japan). After scanning to obtain fluorescence images of the sections, they were imported into ImageJ software and analyzed to obtain quantitative results to represent the average density.

### 2.9 Immunohistochemistry (IHC)

After conventional sectioning, the kidney, duodenum, and femur (5 μm) were subjected to xylene dewaxing, gradient concentration ethanol rehydration, antigen repair, serum blocking, first antibody incubation (*Nrf2* and *HO‐1* antibody 1:100), second antibody incubation, ABC working solution incubation, and microscopic observation. ImageJ software was used to quantify and calculate the average density values for each field of view.

### 2.10 Real‐time reverse transcription (RT)‐polymerase chain reaction (PCR)

After weighing 200 mg of kidney, duodenum, and femur tissues, they were ground into a powder in liquid nitrogen. An RNA extraction kit was used to isolate total RNA and Prime Script RT Master Mix was used for RT. SYBR premixed q‐PCR reagents were used to perform real‐time RT‐PCR on a PCR amplification instrument. Finally, the expression of relative mRNA was quantified by comparing circulating threshold values, with β‐actin as an internal parameter. The primer sequences are listed in Table 1.

**TABLE 1:** Primers for RT‐qPCR.

Gene	Forward sequence (5′‐3′)	Reverse sequence (5′‐3′)
*β‐Actin*	GTGACGTTGACATCCGTAAAGA	GCCGGACACATCGGTACTCC
*Runx2*	GACTGTGGTTACCGTCATGGC	ACTTGGTTTTTCATAACAACAGCGGA
*Bmp2*	GGCCGAAGGTGGATTCTCC	GTCGGGTGTGTTATTGACATACA
*TRPV5*	ACCCTGGCAAGAGTGAAATC	AGTTGGGGTTCCCTAGGTTAT
*CaBP‐9 K*	CGCTAAGAAATCTCCCGAAG	CTCCATCACCGTTCTTATCCA
*PMCA‐1b*	ACTCTGGGGCCAGCTTATTT	GTTCGGCATGGTCAATCTCT

### 2.11 Western blot assay

After weighing 200 mg of kidney, duodenum, and femur tissues, they were ground into a powder in liquid nitrogen. The protein concentration was measured, and the protein was denatured at a high temperature. Denatured protein samples were separated using 10% SDS‐PAGE, transferred to a PVDF membrane, sealed with the sealing solution for 30 min, incubated with the primary antibody overnight at low temperature (Runx2 1:1000, Bmp2 1:1000, transient receptor potential channel V5 [TRPV5] 1:1000, CaBP‐9 k 1: 1000, and PMCA‐1b 1:1000), incubated with the secondary antibody at room temperature for 1 h, and imaged using the Azure Bioimaging System (California, USA). ImageJ software was used to quantify the grayscale values of the stripes, and β‐actin (1:2000) was used as an internal parameter.

### 2.12 Statistical analysis

The data were statistically analyzed using SPSS 25.0, and the results were expressed as mean ± standard deviation (SD) or standard error of mean (SEM). One‐way analysis of variance (ANOVA) was used to identify significant differences, with *p* < .05 indicating a statistically significant difference.

## 3 RESULTS

### 3.1 Successful establishment of DOP model

**FIGURE 3:**
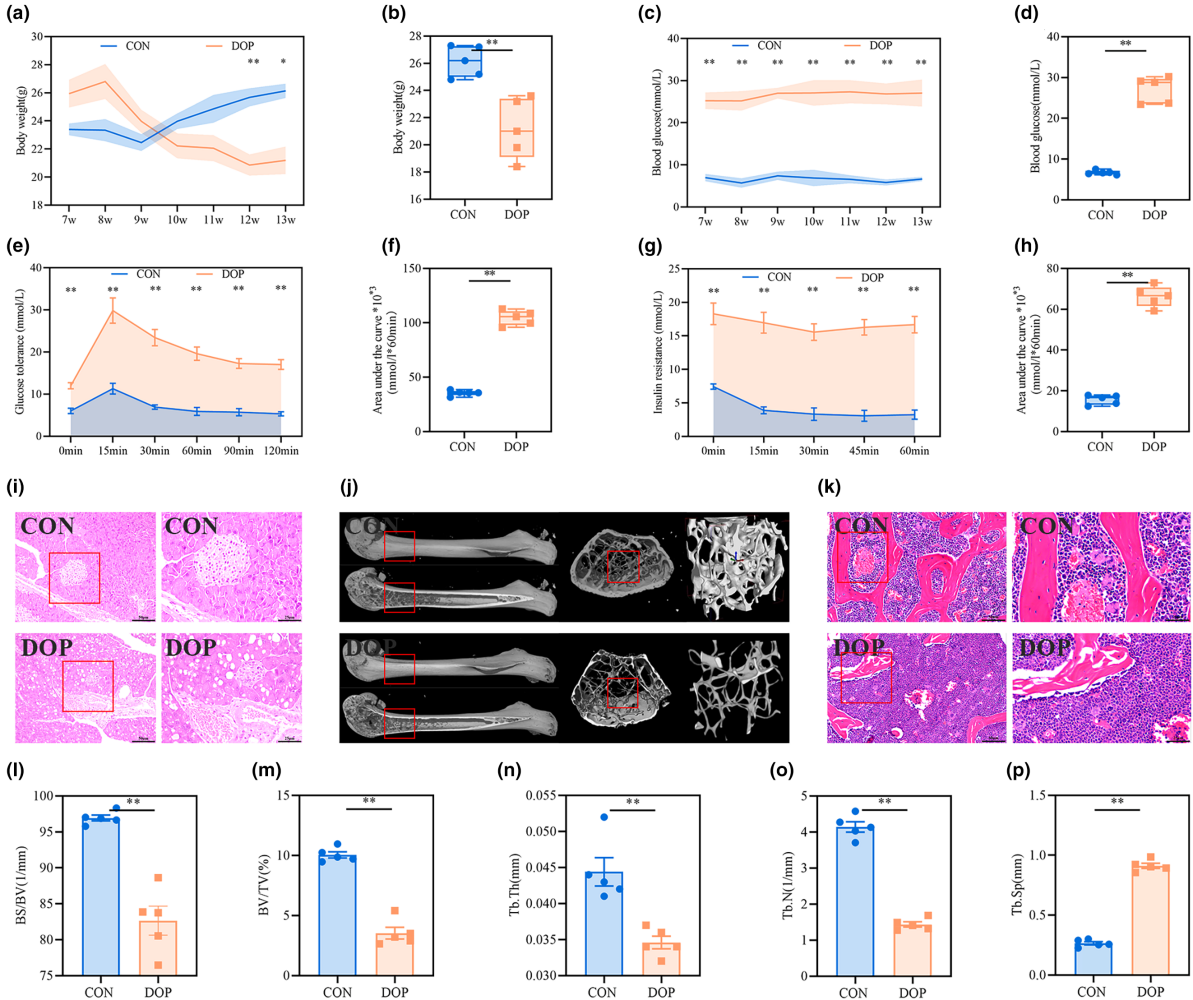
Establishment and identification of DOP model. (a) Body weight during the experiment (*n* = 5). (b) Body weight at the end of the experiment (*n* = 5). (c) Blood glucose levels during the experiment (*n* = 5). (d) Blood glucose levels at the end of the experiment (*n* = 5). (e, f) Glucose tolerance and area under curve analysis (*n* = 5). (g, h) Insulin resistance and area under curve analysis (*n* = 5). (i) Histopathological changes in the pancreas (×400, Scale bar: 50 μm; ×800, Scale bar: 25 μm; *n* = 5). (j) Micro‐CT three‐dimensional reconstruction images of the femoral metaphysis and the related parameter analysis including BS/BV(1/mm) (l), BV/TV (%) (m), Tb. N (1/mm) (n), Tb. Th (mm) (o), and Tb. Sp (mm) (p) (*n* = 5). (k) HE staining image of the femoral shaft epiphysis (×400, Scale bar: 50 μm; ×800, Scale bar: 25 μm; *n* = 5). Data are expressed as mean ± SD or SEM. *p < .05, **p < .01 compared with the DOP group.

The fundamental characteristics of diabetes include continuous hyperglycemia, gradual weight loss, and insulin dysfunction (Tanase et al., 2020). DOP leads to low bone mass, increased risk for fractures, and poor bone health (Khosla et al., 2021). Therefore, we confirmed the success of the model by examining the body weight, blood glucose levels, glucose catabolism, bone mass, and tissue microstructure of the pancreas and femur in the model animal. As shown in Figure 3a,b, the body weight of DOP mice was significantly lower than that of the controls (*p* < .05 or .01). Blood glucose levels in DOP mice remained above 16.7 mmol/L, which were significantly higher than those in the controls (Figure 3c,d, *p* < .01). Compared with the control group, DOP mice showed a rapid increase and a long duration of blood glucose levels after glucose administration, while blood glucose levels decreased slowly after oral insulin administration, always remaining around 16.7 mmol/L, indicating that DOP mice had a reduced tolerance to glucose and insulin (Figure 3e–h, *p* < .01). The pancreatic islets of control mice had a complete and regular morphology and clear cell boundaries, whereas those of DOP mice had a damaged structure and irregular morphology, with a large number of vacuoles were distributed in the cells (Figure 3i). Moreover, we analyzed the bone morphological parameters of the epiphyseal bone in the femur using micro‐CT to examine the alterations in the bone microstructure. As shown in Figure 3j,l–p, the bone mass and bone density of the femurs in DOP mice were significantly decreased, as indicated by the reduced BS/BV, BV/TV, Tb. N, and Tb. Th and increased Tb. Sp (*p* < .01). As illustrated in Figure 3k, HE staining showed that the bone trabeculae in the femoral epiphysis of the control mice were mainly composed of a dense and regular fibrous network. However, the trabeculae in the femoral epiphysis lost their regular reticular structure and became thinner in DOP mice.

### 3.2 Enhancement of bone remodeling ability in DOP mice treated with EUL aqueous extract

**FIGURE 4:**
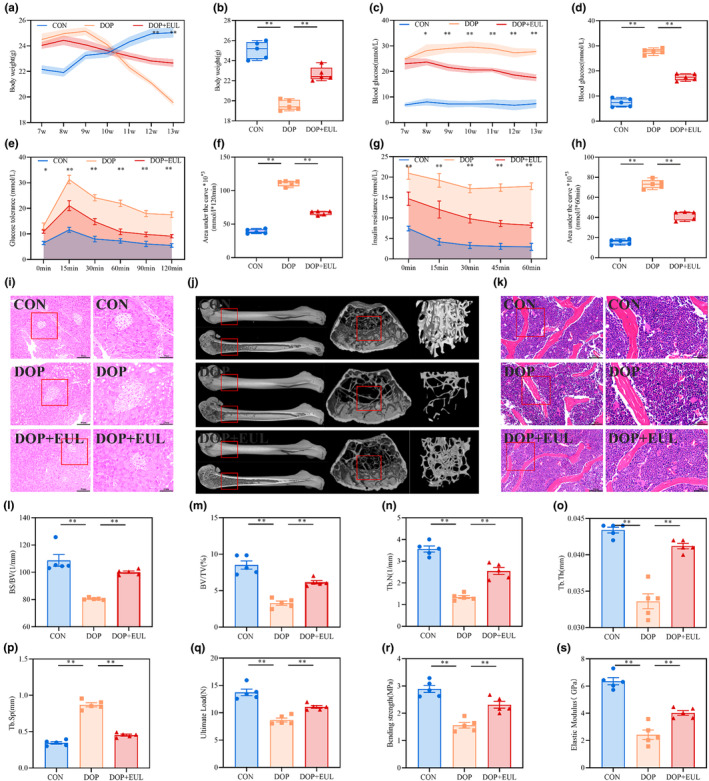
EUL aqueous extract improved the microstructure and strength of the femur in DOP mice. (a) Body weight during the experiment (*n* = 5). (b) Body weight at the end of the experiment (*n* = 5). (c) Blood glucose levels during the experiment (*n* = 5). (d) Blood glucose levels at the end of the experiment (*n* = 5). (e, f) Glucose tolerance and area under curve analysis (*n* = 5). (g, h) Insulin resistance and area under curve analysis (*n* = 5). (i) Histopathological changes in the pancreas (×400, Scale bar: 50 μm; ×800, Scale bar: 25 μm; *n* = 5). (j) Micro‐CT three‐dimensional reconstruction images of the femoral metaphysis and the related parameter analysis including BS/BV(1/mm) (l), BV/TV (%) (m), Tb. N (1/mm) (n), Tb. Th (mm) (o), and Tb. Sp (mm) (p) (*n* = 5). (k) HE staining image of the femoral shaft epiphysis (×400, Scale bar: 50 μm; ×800, Scale bar: 25 μm; *n* = 5). The analysis of the results and related parameters of the femoral three‐point bending test including ultimate load (q), bending strength (r), and elastic modulus (s) (*n* = 5). Data are expressed as mean ± SD or SEM. *p < .05, **p < .01 compared with the DOP group.

As illustrated in Figure 4a–i, DOP mice manifested increased blood glucose levels, decreased body weight and capacity for glucose catabolism, and destruction of pancreatic tissue. However, treatment of DOP mice with EUL aqueous extract decreased the blood glucose level, increased the body weight and capacity for glucose catabolism, and improved the pancreatic tissue structure, characterized by clearing the acinar boundaries and reducing the vacuoles present in the cytoplasm (*p* < .05 or .01). Moreover, as shown in Figure 4jl–p, DOP mice had reduced bone mass and disorganized trabecular alignment of the epiphysis in the femur (Figure 4k). Interestingly, EUL aqueous extract treatment significantly reduced bone loss and retained trabecular bone structures in the femoral epiphysis of DOP mice (*p* < .01). Persistent hyperglycemia also reduces mechanical strength and increases the fracture risk of the bone (Compston, 2018). Therefore, the biomechanical properties of the femurs in different mice were evaluated by subjecting them to a three‐point bending test. As illustrated in Figure 4q–s, the parameters related to the ultimate load, bending strength, and elastic modulus of the femur in DOP mice were significantly lower than those in the controls. Interestingly, the treatment of DOP mice with EUL aqueous extract reversed the reduction in the aforementioned bone strength parameters (*p* < .01). These findings demonstrated that EUL promotes bone remodeling in DOP mice.

### 3.3 Calcium homeostasis in DOP mice was maintained with EUL aqueous extract

**FIGURE 5:**
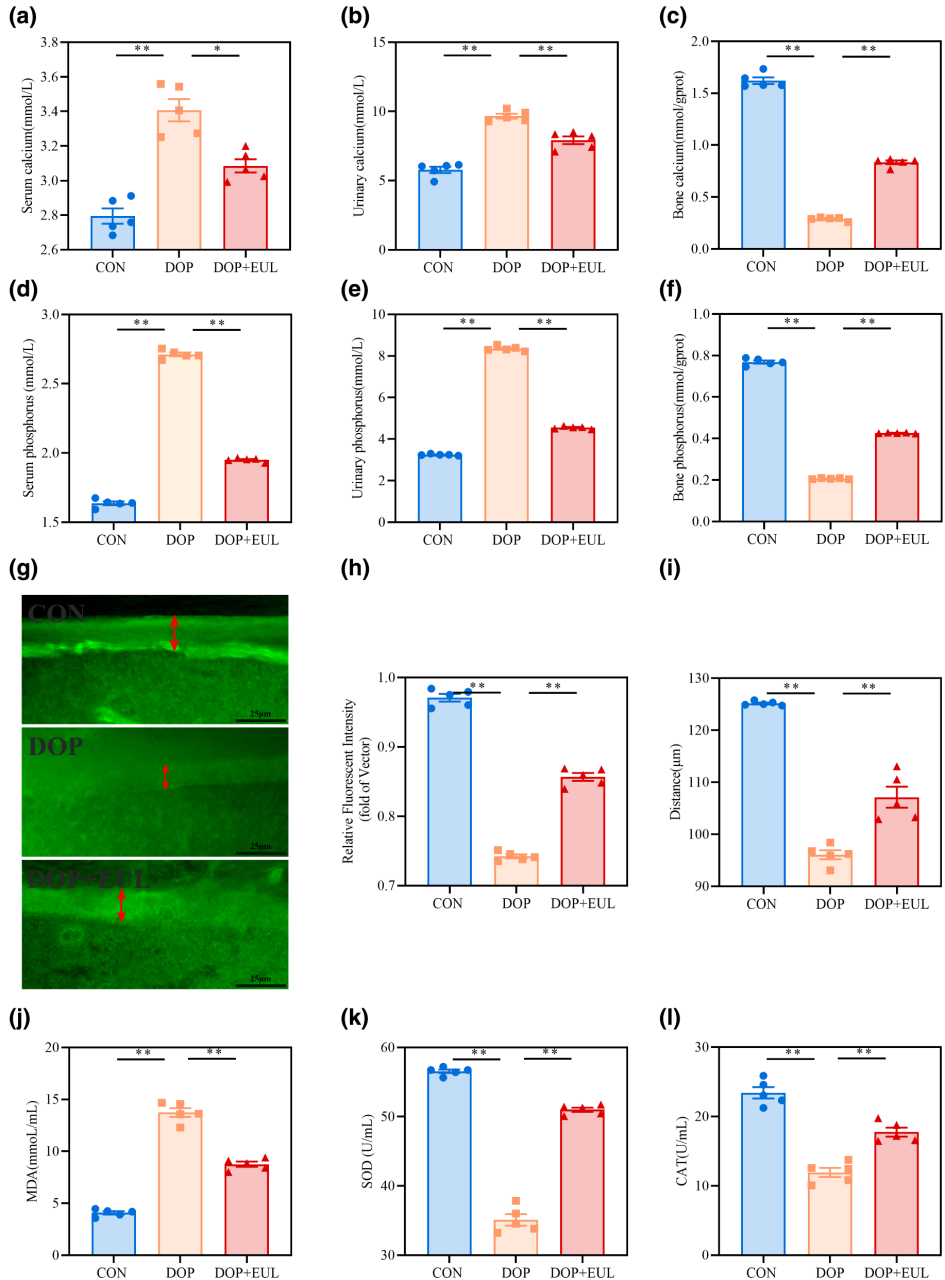
EUL aqueous extract improved calcium metabolism and regulated oxidative stress in DOP mice. (a) Serum Ca^2+^ levels (*n* = 5). (b) Ca^2+^ levels in the urine (*n* = 5). (c) Ca^2+^ levels in the femur (*n* = 5). (d) Serum P^2+^ levels (*n* = 5). (e) P^2+^ levels in the urine (*n* = 5). (f) P^2+^ levels in the femur (*n* = 5). (g) Fluorescent image of bone calcium content and bone formation represented by calcein in the femur (×800, Scale bar: 25 μm; *n* = 5). (h) Analysis of relative fluorescence intensity and distance (i) of the femur (*n* = 5). (j‐l) Serum MDA, SOD, and CAT levels (*n* = 5). Data are expressed as mean ± SD or SEM. *p < .05, **p < .01 compared with the DOP group.

Calcium homeostasis makes contributes to the physiological processes of cellular signal conduction and bone metabolism (Matikainen et al., 2021). Therefore, the effect of EUL aqueous extract on calcium homeostasis in DOP mice was evaluated by measuring Ca^2+^ and P^2+^ levels in the serum, urine, and femurs. As shown in Figure 5a,d, Ca^2+^ and P^2+^ levels in the serum of DOP mice were higher than those in controls (*p* < .01). Meanwhile, Ca^2+^ and P^2+^ levels in the urine of DOP mice were increased compared with those in the controls (Figure 5b,e, *p* < .01). However, Ca^2+^ and P^2+^ levels in the femurs of DOP mice were significantly lower than those in the controls (Figure 5c,f, *p* < .01). Furthermore, we employed calcein for calcium metabolic labeling of the femur and found that the bone mineralization of the femur in DOP mice was notably reduced compared with that in the controls (Figure 5g,h, *p* < .01). Interestingly, EUL aqueous extract treatment restored serum Ca^2+^ and P^2+^ levels, reduced the excretion of urinary Ca^2+^and P^2+^, and increased the content of bone Ca^2+^and P^2+^ in the DOP mice (*p* < .05 or .01).

### 3.4 Calcium homeostasis was regulated by inhibition of oxidative stress treated with EUL aqueous extract

The impaired calcium homeostasis in patients with diabetes is mainly attributed to an oxidative stress imbalance (Naidoo et al., 2022). Oxidative stress affects bone metabolism by interfering with the balance between osteoclast and osteoblast activity or the calcium absorption function of the intestinal and kidneys, which can lead to calcium disorders (Rivoira et al., 2015; Zhu et al., 2022). The *Nrf2*/*HO‐1* signaling pathway detects early oxidative stress and induces antioxidant responses, and is instrumental in regulating the oxidative and antioxidant balance (An et al., 2021).

#### 3.4.1 Effects of EUL aqueous extract on oxidative stress in DOP mice

Oxidative stress is an important mechanism that leads to DOP. As illustrated in Figure 5j–l, SOD and CAT levels in the serum of DOP mice were significantly reduced, lowering than those in the controls (*p* < .01). However, the serum MDA levels in DOP mice were significantly higher than those in the controls (*p* < .01). Interestingly, after 6 weeks of EUL aqueous extract administration, the serum levels of SOD and CAT were significantly increased (*p* < .01). Meanwhile, the serum MDA levels decreased (Figure 5j–l, *p* < .01). These results indicated that EUL has antioxidative stress function in DOP mice.

#### 3.4.2 The balance of osteoclast and osteoblast activity was regulated by activating the *Nrf2/HO‐1* pathway in DOP mice with EUL aqueous extract

**FIGURE 6:**
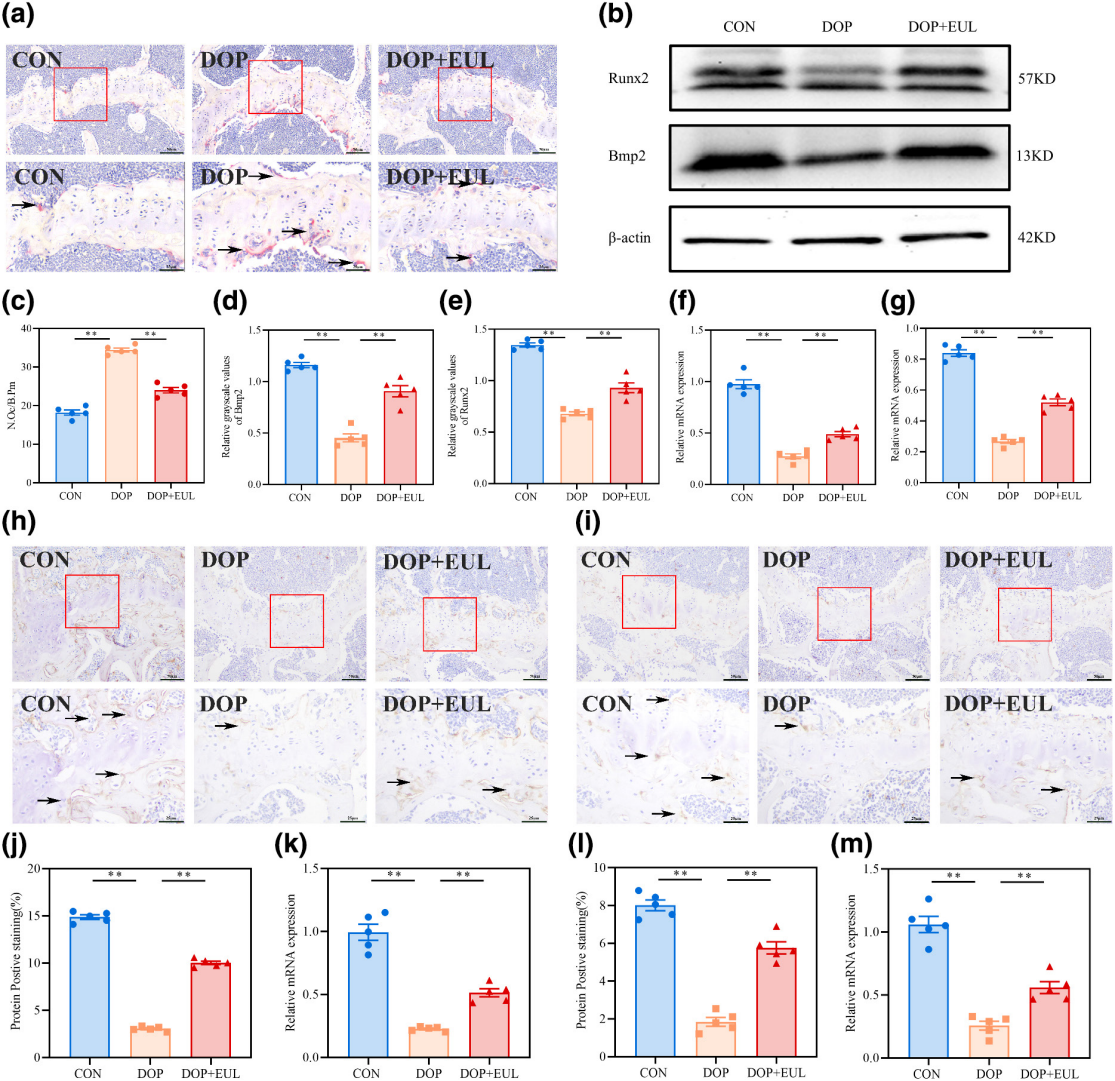
EUL aqueous extract improved bone metabolism by activating the *Nrf2*/*HO‐1* pathway in DOP mice. (a) TRAP staining images (black arrow; ×400, Scale bar:50 μm; ×800, Scale bar:25 μm) and analysis (c) of femoral metaphysis (*n* = 5). (b) Western blot expression of β‐actin, Bmp2, Runx2 and analysis of Bmp2 (d) and Runx2 (e) in the femur tissue (*n* = 5). The mRNA expressions of *Bmp2* (f) and *Runx2* (g) were analyzed using RT‐qPCR (*n* = 5). (h) *Nrf2* IHC staining image (black arrow; ×400, Scale bar:50 μm; ×800, Scale bar:25 μm) in the femur and the semi‐quantitative analysis (j) (*n* = 5). (i) *HO‐1* of IHC staining image (black arrow; ×400, Scale bar:50 μm; ×800, Scale bar:25 μm) in the femur and the semi‐quantitative analysis (l) (*n* = 5). Analysis of mRNA expressions of *Nrf2* (k) and *HO‐1* (m) in the femur using RT‐qPCR (*n* = 5). Data are expressed as mean ± SD or SEM. **p < .01 compared with the DOP group.

Bone metabolism actively participates in the maintenance of calcium homeostasis, primarily through the interaction between bone formation and absorption (Zhang et al., 2011). Oxidative stress disrupts bone metabolic homeostasis by increasing osteoblast apoptosis and inhibiting bone mineralization and osteogenesis, while promoting osteoclast formation and exacerbating bone resorption (Zhu et al., 2022). Bone absorption and mineral release by osteoclasts are important factors associated with bone related diseases (Rathinavelu et al., 2018). Bmp2 and Runx2 are important osteoblast transcription factors that play essential roles in bone formation (Fatchiyah et al., 2021). As shown in Figure 6a,c, a marked increase in osteoclasts number was observed in the femurs of DOP mice compared with that in the controls (*p* < .01). Bmp2 and Runx2 expression in the femurs of DOP mice was notably reduced (Figure 6b,d–g, *p* < .01). Furthermore, the speed of dynamic bone formation in DOP mice was significantly decreased (Figure 5g,i, *p* < .01). However, after 6 weeks of EUL aqueous extract administration, the number of osteoclasts was reduced and Bmp2 and Runx2 expression was significantly enhanced in femurs of DOP mice (*p* < .01). Moreover, EUL aqueous extract treatment significantly improved bone metabolism and promoted bone formation (*p* < .01).

Next, we assessed *Nrf2* and *HO‐1* expressions in the femurs of DOP mice using RT‐qPCR and IHC staining to examine the effect of the EUL aqueous extract on oxidative stress. As illustrated in Figure 6h–m, a remarkable reduction in *Nrf2* and *HO‐1* expressions was observed in the femurs of DOP mice compared with that in the controls (*p* < .01). However, treatment with the EUL aqueous extract significantly upregulated *Nrf2* and *HO‐1* expressions in DOP mice. Overall, these results suggested that the EUL aqueous extract may upregulate the *Nrf2/HO‐1* pathway expressions in DOP mice to balance bone metabolic activity.

#### 3.4.3 Calcium absorption in DOP mice was promoted through *Nrf2*/*HO‐1* activation of EUL aqueous extract to regulate the *TRPV5/CaBP‐9k/PMCA‐1b* pathway

**FIGURE 7:**
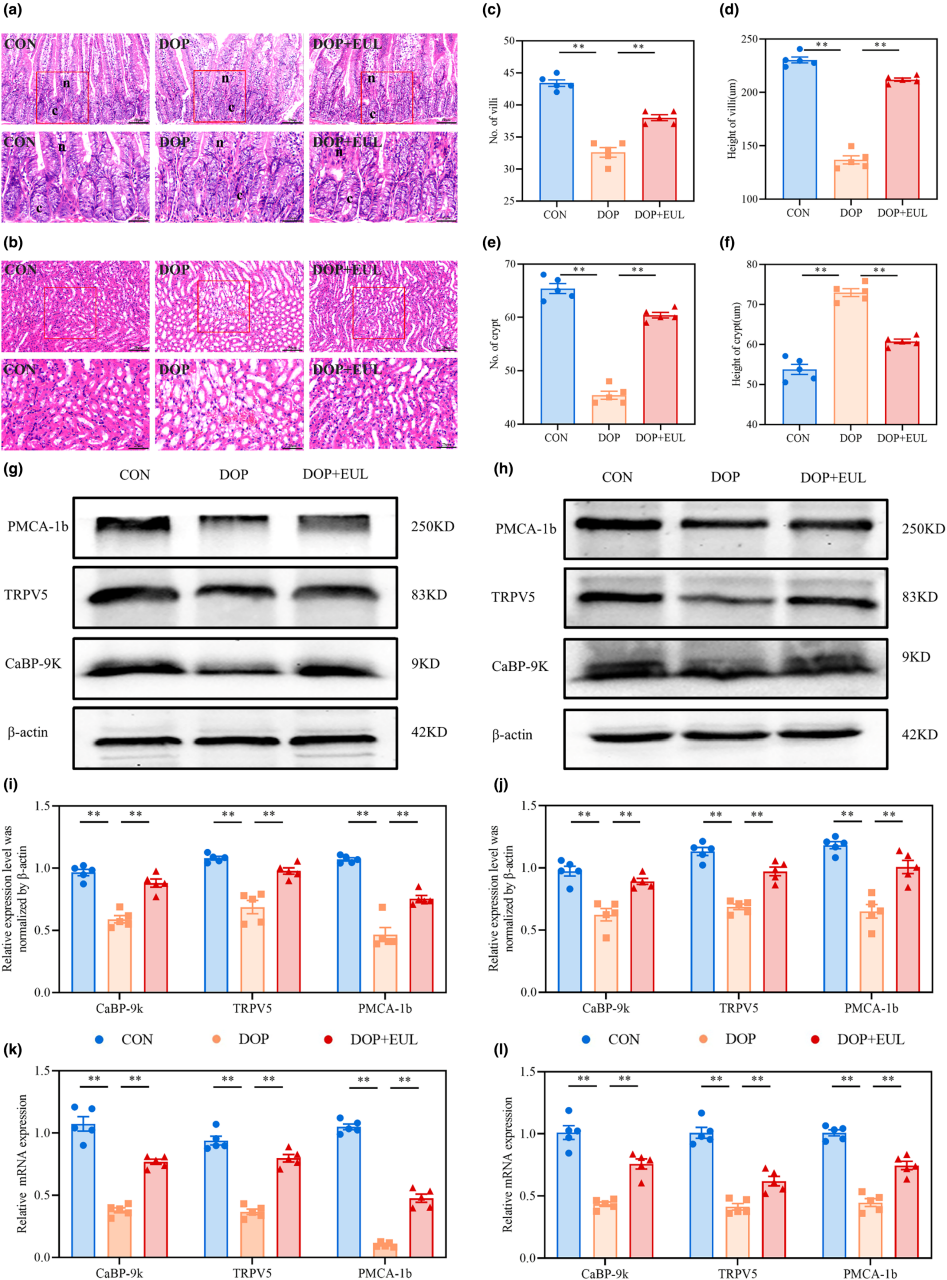
EUL aqueous extract promotes intestinal calcium absorption and inhibits renal calcium loss by regulating the *TRPV5/CaBP‐9 k/PMCA‐1b* pathway. (a) HE staining images of small intestine tissue (n: Villus, c: Crypt; ×400, Scale bar: 50 μm; ×800, Scale bar: 25 μm) and their analysis: Number of intestinal villi (c), height of intestinal villus (d), number of intestinal crypts (e), and height of intestinal crypts (f) (*n* = 5). (b) HE staining images of the renal tubule (×400, Scale bar: 50 μm; ×800, Scale bar: 25 μm; *n* = 5). (g) Western blot images and analysis (i) of the small intestinal calcium channel proteins TRPV5, CaBP‐9 k, and PMCA‐1b (*n* = 5). (h) Western blot images and analysis (j) of the renal calcium channel proteins TRPV5, CaBP‐9 k, and PMCA‐1b (*n* = 5). (k) The mRNA expression of *TRPV5*, *CaBP‐9 k*, and *PMCA‐1b* in the small intestine was analyzed using RT‐qPCR (*n* = 5). (l) The mRNA expression of *TRPV5*, *CaBP‐9 k*, and *PMCA‐1b* in the kidneys was analyzed using RT‐qPCR (*n* = 5). Data are expressed as mean ± SD or SEM. **p < .01 compared with the DOP group.

The integrity of intestinal morphology and structure of the intestine directly affects many diseases and intestinal calcium absorption (Lee & Kim, 2018). The renal tubules play a leading role in the excretion and reabsorption functions of the kidney, affecting renal calcium reabsorption (Duan et al., 2021). As illustrated in Figure 7a,c–f, the mucosal necrotic areas in DOP mice were remarkably increased, even when exfoliated, compared with those in the controls. Moreover, the height and structure of the small intestinal villi were impaired, the brush border became discontinuous, and inflammatory cells infiltrated. The depth of the small intestinal gland recess significantly increased, leading to a decrease in the potential for differentiation (*p* < .01). In addition, as shown in Figure 7b, the renal tubules of DOP mice exhibited dilation, vacuolar degeneration in the cytoplasm, and apoptosis in some cells. The aqueous extract of EUL remarkably ameliorated the structure of intestinal and renal tissues in DOP mice and alleviated their pathological changes.

The Ca^2+^ transcellular pathway involves Ca^2+^ passing through TRPV5 and TRPV6 on the cell membrane and entering the cytoplasm in a paraelectric chemical gradient to bind to calcium‐binding proteins (CaBP‐9 k). Ca^2+^ entering the cytoplasm is transported to the cell basement membrane. Subsequently, Ca^2+^ is transported to the blood via calcium pumps (PMCA‐1b and NCX1) in the cell basement membrane (Areco et al., 2020). Therefore, we examined the expressions of calcium transporters using western blotting and assessed the expression of calcium transporter genes using RT‐qPCR in the intestinal of DOP mice. As shown in Figure 7g,i,k, the expression levels of TRPV5, CaBP‐9 K, and PMCA‐1b were lower in DOP mice than those in the controls (*p* < .01). Renal western blotting and RT‐qPCR results revealed that TRPV5, CaBP‐9 K, and PMCA‐1b expression was significantly reduced in the kidneys of DOP mice (Figure 7h,j,l, *p* < .01). Interestingly, administration of the EUL aqueous extract significantly upregulated the expression of the intestinal and renal calcium transport channels TRPV5, CaBP‐9 k, and PMCA‐1 in DOP mice (*p <* .01).

**FIGURE 8:**
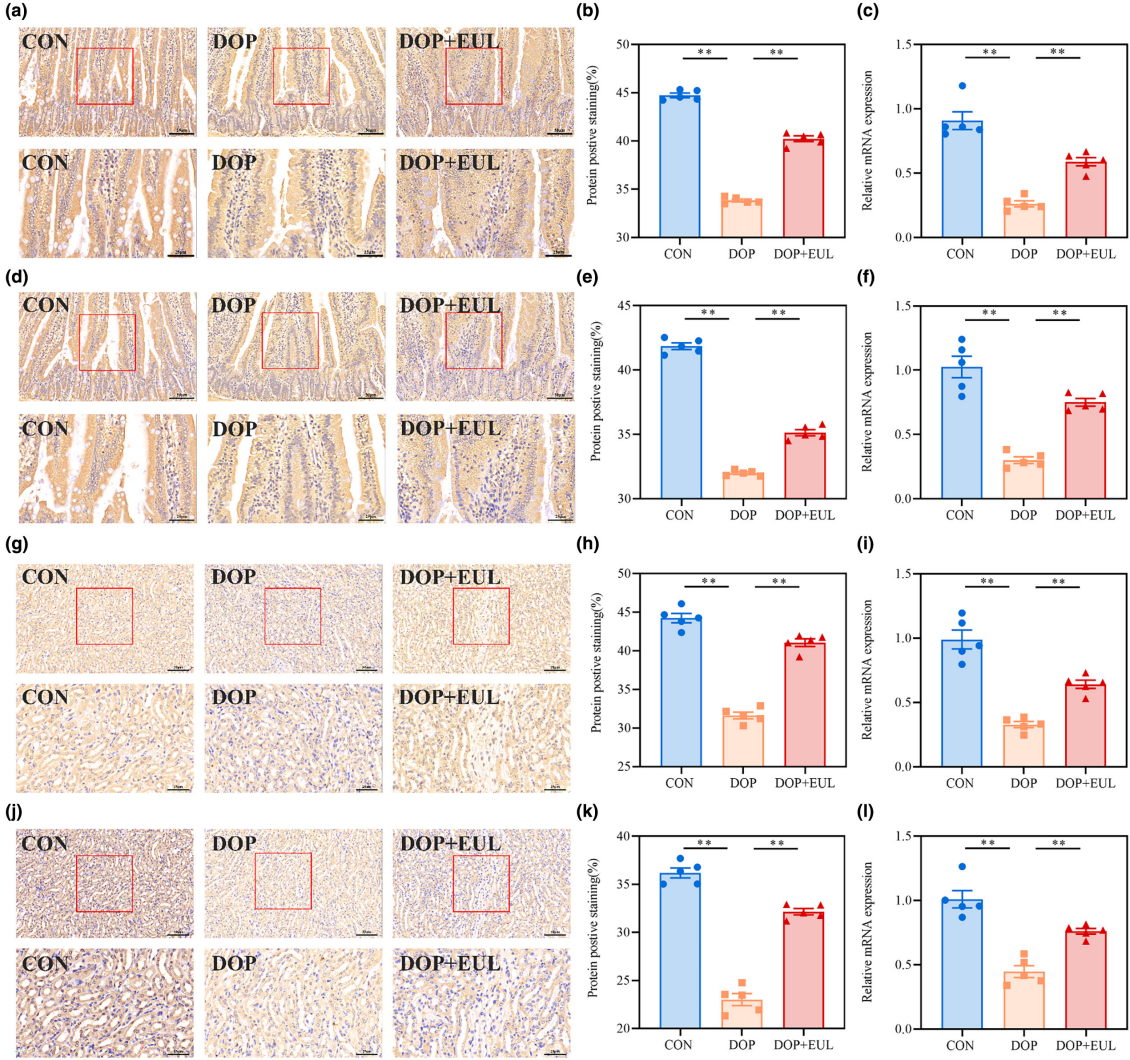
EUL aqueous extract treatment improves oxidative stress by regulating the *Nrf2*/*HO‐1* signaling pathway in the small intestine and kidney of DOP mice. (a) IHC staining image of *Nrf2* in small intestine (×400, Scale bar:50 μm; ×800, Scale bar:25 μm) and semi‐quantitative analysis (b) (*n* = 5). (c) The mRNA expression of *Nrf2* in the small intestine was analyzed using RT‐qPCR (*n* = 5). (d) IHC staining image of *HO‐1* in the small intestine (×400, Scale bar:50 μm; ×800, Scale bar: 25 μm) and semi‐quantitative analysis (e) (*n* = 5). (f) The mRNA expression of *HO‐1* in the small intestine was analyzed using RT‐qPCR (*n* = 5). (g) IHC staining image of *Nrf2* in the kidney (×400, Scale bar:50 μm; ×800, Scale bar: 25 μm) and semi‐quantitative analysis (h) (*n* = 5). (i) The mRNA expression of *Nrf2* in the kidney was analyzed using RT‐qPCR (*n* = 5). (j) IHC staining image of *HO‐1* in the kidney (×400, Scale bar:50 μm; ×800, Scale bar: 25 μm) and semi‐quantitative analysis (k) (*n* = 5). (l) The mRNA expression of *HO‐1* in the kidney was analyzed using RT‐qPCR (*n* = 5). Data are expressed as mean ± SD or SEM. **p < .01 compared with the DOP group.

Moreover, we assessed *Nrf2* and *HO‐1* expressions in the intestinal and kidney of DOP mice using RT‐qPCR and IHC staining to examine the effect of the EUL aqueous extract on oxidative stress. As illustrated in Figure 8a–f, a remarkable reduction in *Nrf2* and *HO‐1* expression was observed in the small intestine of DOP mice was observed compared with that in the controls (*p* < .01). Meanwhile, as displayed in Figure 8g–l, we discovered that *Nrf2* and *HO‐1* expression in the kidneys of DOP mice was also significantly reduced compared with that in the controls (*p* < .01). Notably, treatment with EUL aqueous extract significantly upregulated the *Nrf2* and *HO‐1* expression in DOP mice. Overall, these results suggested that the EUL aqueous extract may upregulate the *Nrf2*/*HO‐1* pathway in DOP mice to increase the levels of TRPV5/CaBP‐9 k/PMCA‐1b calcium transport channel (*p* < .01), which improves intestinal calcium and renal calcium absorption.

### 3.5 Favorable biosafety profile of EUL aqueous extract in DOP mice

**FIGURE 9:**
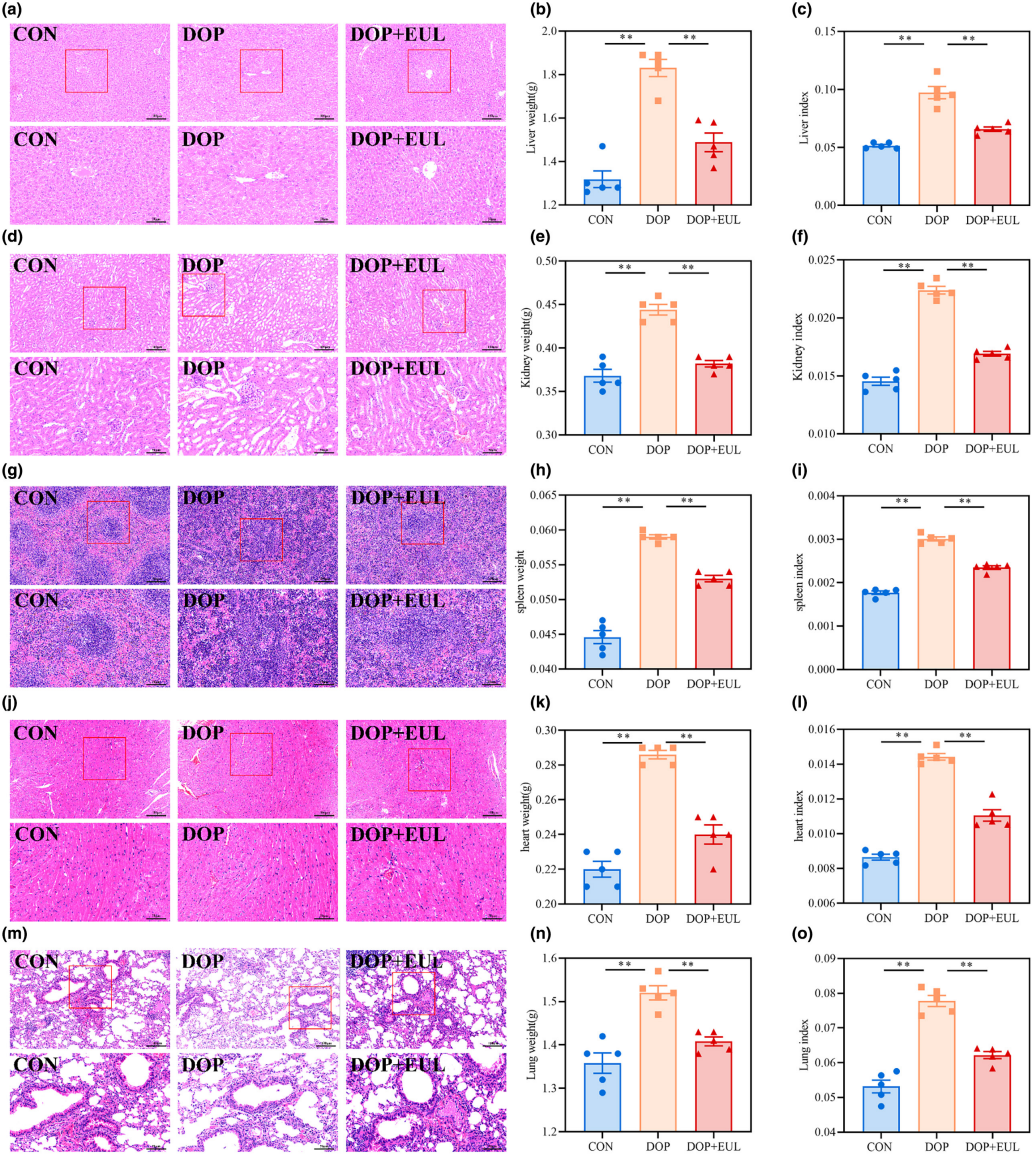
EUL aqueous extract improves histopathological changes in the liver, kidney, spleen, heart, and lungs of DOP mice. (a) Liver HE staining representative images (×200, Scale bar: 100 μm; ×400, Scale bar: 50 μm; *n* = 5). (b) Liver weight (*n* = 5). (c) Liver index (liver weight/body weight) (*n* = 5). (d) Kidney HE staining representative images (×200, Scale bar: 100 μm; ×400, Scale bar: 50 μm; *n* = 5). (e) Kidney weight (*n* = 5). (f) Kidney index (kidney weight/body weight) (*n* = 5). (g) Spleen HE staining representative image (×200, Scale bar: 100 μm; ×400, Scale bar: 50 μm; *n* = 5). (h) Spleen weight (*n* = 6). (i) Spleen index (spleen weight/body weight) (*n* = 5). (j) Heart HE staining representative image (×200, Scale bar: 100 μm; ×400, Scale bar: 50 μm; *n* = 5). (k) Heart weight (*n* = 6). (l) Heart index (heart weight/body weight) (*n* = 5). (m) Lung HE staining representative images (×200, Scale bar: 100 μm; ×400, Scale bar: 50 μm; *n* = 5). (n) Lung weight (*n* = 5). (o) Lung index (lung weight/body weight) (*n* = 5). Data are expressed as mean ± SD or SEM. **p < .01 compared with DOP group.

To study the toxic effects of the aqueous extract of EUL, the microstructures of the liver, kidney, spleen, heart, and lungs were observed. HE staining of liver tissue is shown in Figure 9a. HE staining showed that compared with the controls, the hepatic cords of DOP mice were disordered, with swollen and degenerated hepatocytes and vacuolar degeneration. Moreover, as shown in Figure 9b,c, the liver weight and liver index of DOP mice increased significantly (*p* < .01). As shown in Figure 9d, HE staining of the kidneys in DOP mice revealed hypertrophic glomeruli, irregular shape, cytoplasmic vacuolar degeneration, thickened basement membrane, irregular arrangement of renal tubules, and dilation and deformation. Moreover, as illustrated in Figure 9e,f, the renal weight and index of DOP mice increased significantly compared with those of the controls (*p* < .01). As illustrated in Figure 9g, HE staining of the spleen showed atrophy of the splenic tegument, disorganization of the red and white medullary structures, and a decrease in the number of lymphocytes in DOP mice compared with that in the control group. Meanwhile, as shown in Figure 9h,i, the spleen weight and index of DOP mice increased significantly compared with those of the controls (*p* < .01). As shown in Figure 9j, HE staining of the heart revealed that the cardiac muscle fibers in DOP mice were disorganized and sparse, with intercellular vacuoles and enlarged gaps. Moreover, as shown in Figure 9k,l, the heart weight and index of DOP mice were increased significantly (*p* < .01). As shown in Figure 9m, HE staining of lung tissue revealed thickening of the alveolar walls, a significant increase in the thickness of the alveolar septa, and infiltration of inflammatory cells in the interstitium of the lungs in DOP mice. Furthermore, as shown in Figure 9n,o, the lung weight and lung index of DOP mice were increased significantly (*p* < .01). Interestingly, liver, kidney, spleen, heart, and lung damage in DOP mice was significantly ameliorated after treatment with the aqueous extract of EUL (*p* < .01). These results demonstrated that the aqueous extract of EUL was biologically safe.

## 4 DISCUSSION

DM is a chronic disease caused by hyperglycemia that can lead to many secondary diseases. Persistent hyperglycemia can severely impair bone health and promote the onset and progression of DOP (Ma et al., 2020; Sumida et al., 2020). The prevalence of DOP has increased in recent years. Therefore, it is crucial to explore new approaches for the treatment of DOP. Recent studies have shown that oxidative stress and calcium homeostasis play an important role in maintaining bone health (Naidoo et al., 2022). EUL, a traditional Chinese medicine, significantly improves bone quality (Huang et al., 2021; Hussain et al., 2016). The current study demonstrated several novel findings and suggested that the EUL aqueous extract ameliorates bone loss in DOP mice by inhibiting oxidative damage‐mediated disturbances in calcium homeostasis via increased expression of *Nrf2/HO‐1*. To the best of our knowledge, this is the first proof of an osteoprotective effect under diabetic conditions. Modulation of the *Nrf2/HO‐1* pathway maintained calcium homeostasis by upregulating the expression of *TRPV5/CaBP‐9 K/PMCA‐1b* and improving bone metabolism. Specifically, diabetes‐induced oxidative stress altered the expression of calcium channel transporters and disrupted the balance between osteogenic and osteoblastic activities, leading to disturbed calcium homeostasis. The inhibition of oxidative stress in DOP mice maintained calcium homeostasis and subsequently restored bone formation.

EUL contains various active substances and has been used to treat osteoporosis for more than 3000 years (Huang et al., 2021). Pan et al. (2014) reported that EUL effectively prevented hindlimb suspension‐induced bone loss. Qi et al. (2019) also found that EUL extract has protective effects on both the stimulation of bone formation and the suppression of bone resorption in lead‐exposed rats. However, the protective effects of EUL on diabetes‐induced bone loss have not yet been studied. In the present study, we demonstrated that EUL aqueous extract alleviated diabetic symptoms, increased femoral bone strength, and improved bone microarchitecture in DOP mice. Administration of the EUL aqueous extract also decreased the number of osteoclasts and promoted the expression of osteogenesis‐related factors in DOP mice. Therefore, we concluded that the EUL aqueous extract has a strong anabolic function in bone formation in a DOP mice model.

Oxidative stress is closely associated with the development and complications of DM. Nrf2 is a transcription factor regulating oxidative stress (Bian et al., 2022). HO‐1, an Nrf2‐regulated cytoprotective molecule, is particularly important in protecting cells from oxidative damage (Saha et al., 2020). In previous studies, the *Nrf2/HO‐1* signaling pathway alleviated pathological damage to organs, such as the femur, intestine, kidney, liver, and heart, by antagonizing oxidative stress (Do et al., 2018; Ma et al., 2020; Savic et al., 2024; Wu et al., 2023; Xu et al., 2024). However, the effects of the aqueous extract of EUL on multiple organs in the diabetic state have not yet been reported. Our results showed that the expression of *Nrf2* and *HO‐1* was significantly reduced in the small intestine, kidney, and femur of DOP mice, whereas the aqueous EUL extract significantly alleviated their oxidative effects. A novel finding of the current study was that the aqueous extract of EUL alleviated morphological and histopathological alterations in the heart, liver, spleen, lungs, and kidneys of DOP mice.

Calcium and phosphorus are the main mineral elements in the bone and play an important role in bone development and mineralization. Calcium‐phosphorus deficiency or calcium‐phosphorus imbalance during the growth period negatively affects bone maturation and increases the risk of osteoporosis and fractures (Ciosek et al., 2021). The *Nrf2/HO‐1* pathway regulates calcium and phosphorus levels to combat cardiovascular diseases (Lu et al., 2023; Zhang et al., 2020). However, the role of the *Nrf2/HO‐1* pathway in the treatment of DOP by regulating calcium and phosphorus levels has rarely been reported. In the present study, we found that DOP mice had high blood and urine calcium and phosphorus levels and low bone calcium and phosphorus levels. After 6 weeks of oral administration of the aqueous extract of EUL, blood and urine calcium and phosphorus levels were stabilized and femoral calcium and phosphorus levels were improved in DOP mice. This suggested that the aqueous extract of EUL effectively improved bone quality by regulating calcium and phosphorus levels in DOP mice. In addition, the results of a previous study showed that hyperglycemia decreases calcium levels in the serum and bone, while increasing calcium levels in the urine, which leads to insufficient calcium absorption in the body and impairs bone quality. (Yu et al., 2015). The results of the current study are not consistent with this finding, which may be due to the fact that hypocalcemia in the early stages of DM promotes the formation of osteoclasts in the bone tissue, accelerates bone resorption activity, and facilitates the flow of bone calcium into the bloodstream, which ultimately leads to temporary hypercalcemia (Tinawi, 2021). For further validation, calcium staining was performed, and the results confirmed a significant reduction in the calcium levels in the femurs of DOP mice. This suggests that in the diabetic state, calcium absorption in the body changes constantly over time.

The body's calcium requirements depend on calcium homeostasis, which is largely dependent on the function of calcium‐regulating organs such as intestinal absorption, renal reabsorption, and bone metabolism. Intestinal calcium absorption and renal calcium reabsorption constitute the total amount of calcium in the body, and bone metabolism regulates calcium balance in the body (Matikainen et al., 2021; Weaver & Peacock, 2011, 2019). Persistent hyperglycemia damages the structure and function of calcium regulatory organs in the body, resulting in inadequate calcium absorption, which eventually damages bone quality and reduces bone strength (Wongdee et al., 2017). Bone metabolism is an important regulator of calcium homeostasis and occurs via interactions between osteoblasts and osteoclasts (Luo et al., 2021). Hyperglycemia destroys the bone by stimulating osteoclast formation, promoting bone resorption, and releasing the mineral matrix (Rathinavelu et al., 2018). Moreover, persistent hyperglycemia reduces osteoblast proliferation and differentiation by decreasing the expression of osteoblast‐associated factors (Jiao et al., 2015). Intestinal calcium absorption and renal calcium reabsorption are essential components of calcium homeostasis and play critical roles in calcium maintenance (Kasozi et al., 2018; Lee & Kim, 2018). The intestinal mucosal structure constitutes the physical barrier of the intestinal epithelium and is instrumental in intestinal calcium absorption (Diaz de Barboza et al., 2017). Hyperglycemia restricts intestinal calcium absorption by disrupting the structural integrity of the intestinal mucosa and enhancing intestinal permeability (Zhong et al., 2016). Moreover, sustained hyperglycemia interferes with the regulation of low intestinal plasma calcium levels by decreasing calcitonin levels and downregulating intestinal calcium transporter protein expression (Moe, 2016). The kidneys maintain calcium homeostasis through filtration and reabsorption in the renal tubules. TRPV5, CaBP‐9 K, and PMCA‐1b are the active calcium transport channels in the renal tubules that mediate calcium reabsorption in the kidney. Downregulation of renal calcium transport proteins in diabetes results in decreased calcium reabsorption by renal tissues, leading to increased urinary calcium levels and poor prognosis (Liu et al., 2018; Moe, 2016). In this study, DOP mice showed decreased femoral Bmp2 and Runx2 expression and increased osteoclast numbers, suggesting that bone resorption was superior to bone formation. In addition, the intestinal mucosa and renal tubular structures were damaged in DOP mice, with epithelial necrosis in some areas of the small intestinal mucosa, vacuolization of the renal tubular epithelium, and an enlarged lumen. TRPV5, CaBP‐9 K, and PMCA‐1b expression was downregulated in the small intestine and renal calcium transport channels of DOP mice. However, the aqueous extract of EUL upregulated the expression of Bmp2 and Runx2 in the femurs of DOP mice and reduced the number of osteoclasts. Moreover, the aqueous extract of EUL alleviated histopathological changes in the small intestine and kidneys of DOP mice and increased TRPV5, CaBP‐9 K, and PMCA‐1b expression.

**FIGURE 10:**
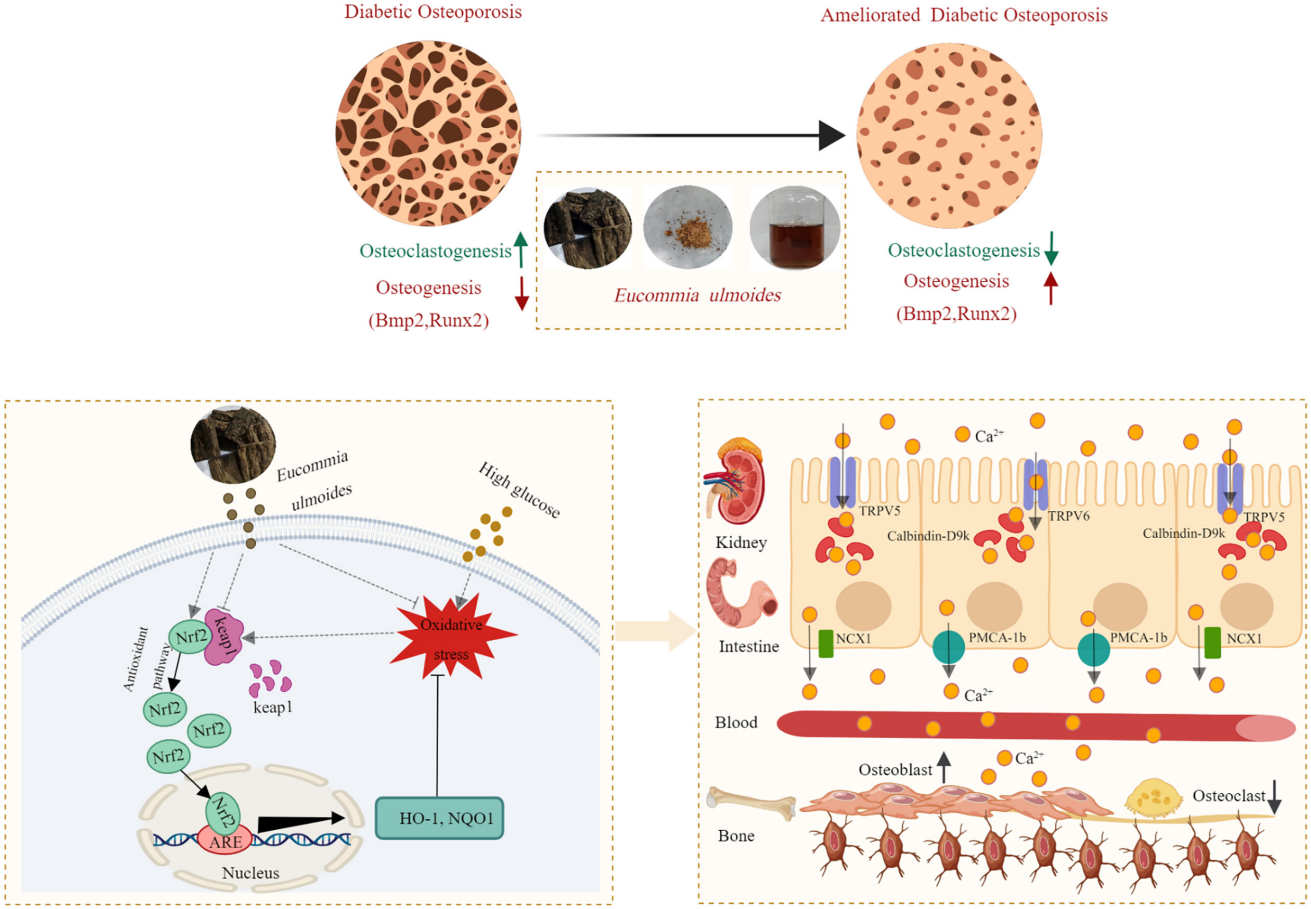
The schematic diagram illustrates the underlying mechanism of EUL aqueous extract in ameliorating bone mass in DOP mice. Administration of EUL aqueous extract in DOP mice eliminates oxidative stress in the body by regulating the *Nrf2/HO‐1* pathway. This promoted *TRPV5/CaBP‐D9k/PMCA‐1b* signaling in the intestine and kidney, which maintained calcium homeostasis in DOP mice and ultimately ameliorated DOP.

The data of this study suggested that the aqueous extract of EUL regulates oxidative stress through the *Nrf2/HO‐1* pathway, which leads to the maintenance of calcium homeostasis through the *TRPV5/CaBP‐D9k/PMCA‐1b* pathway, resulting in improved bone quality, and ultimately ameliorated DOP (Figure 10). However, this study had some limitations. This study did not verify the effect of the aqueous extract of EUL on calcium homeostasis and bone balance in osteoporotic diabetic mice at the cellular level. Whether the specific active ingredients of EUL can ameliorate DOP by regulating calcium homeostasis in the intestines, kidneys, and bones requires further exploration.

## 5 CONCLUSION

Our results suggested that diabetes inhibited *Nrf2/HO‐1* expression, exacerbating oxidative stress, which subsequently exacerbated the dysregulation of calcium homeostasis and triggered bone loss. EUL aqueous extract may inhibit oxidative stress by upregulating the expression of *Nrf2/HO‐1*. In addition, EUL aqueous extract could promote *TRPV5/CaBP‐9 k/PMCA‐1b* signaling and regulate calcium absorption in the intestine and kidney. This restored bone formation and regulated bone metabolic activity. These results demonstrated the usefulness and potential of the EUL aqueous extracts in ameliorating DOP. Whether the specific active ingredients of EUL can ameliorate DOP by regulating calcium homeostasis in the intestines, kidneys, and bones requires further exploration.

## AUTHOR CONTRIBUTIONS

**Jie Shen:** Writing – original draft (lead). **Yichen Gao:** Writing – original draft (equal). **Yuyao Deng:** Methodology (equal). **Zhaoxin Xia:** Project administration (equal). **Xia Wang:** Data curation (equal). **Xianyi He:** Methodology (equal). **Yun He:** Writing – review and editing (equal). **Binbin Yang:** Writing – review and editing (equal).

## CONFLICT OF INTEREST STATEMENT

The authors declare that the research was conducted in the absence of any commercial or financial relationships that could be construed as a potential conflict of interest.

## ETHICS STATEMENT

The animal study was reviewed and approved by the Institutional Ethics Committee of the Affiliated Stomatological Hospital, Southwest Medical University (certificate number, 20221125‐015) and conducted in accordance with the Declaration of Helsinki and its later amendments or comparable ethical standards.

## DECLARATION

All claims expressed in this article are solely those of the authors and do not necessarily represent those of their affiliated organizations, or those of the publisher, the editors, and the reviewers. Any product that may be evaluated in this article, or claim that may be made by its manufacturer, is not guaranteed or endorsed by the publisher.

## Data Availability

The original contributions presented in the study are included in the article. Further inquiries can be directed to the corresponding authors.

## References

[fsn34413-bib-0001] An, R. , Li, D. , Dong, Y. , She, Q. , Zhou, T. , Nie, X. , Pan, R. , & Deng, Y. (2021). Methylcobalamin protects melanocytes from H_2_O_2_‐induced oxidative stress by activating the Nrf2/HO‐1 pathway. Drug Design, Development and Therapy, 15, 4837–4848. 10.2147/DDDT.S336066 34876806 PMC8643160

[fsn34413-bib-0002] Areco, V. A. , Kohan, R. , Talamoni, G. , de Talamoni, N. G. T. , & López, M. E. P. (2020). Intestinal Ca ^2+^ absorption revisited: A molecular and clinical approach. World Journal of Gastroenterology, 26(24), 3344–3364. 10.3748/wjg.v26.i24.3344 32655262 PMC7327788

[fsn34413-bib-0003] Bian, Y. , Yu, H. , Jin, M. , & Gao, X. (2022). Repigmentation by combined narrow‐band ultraviolet B/adipose‐derived stem cell transplantation in the mouse model: Role of Nrf2/HO‐1‐mediated Ca ^2+^ homeostasis. Molecular Medicine Reports, 25(1), 6. 10.3892/mmr.2021.12522 34751412 PMC8600419

[fsn34413-bib-0056] Ciosek, Ż. , Kot, K. , Kosik‐Bogacka, D. , Łanocha‐Arendarczyk, N. , & Rotter, I. (2021). The effects of calcium, magnesium, phosphorus, fluoride, and lead on bone tissue. Biomolecules, 11(4), 506. 10.3390/biom11040506 33800689 PMC8066206

[fsn34413-bib-0005] Compston, J. (2018). Type 2 diabetes mellitus and bone. Journal of Internal Medicine, 283(2), 140–153. 10.1111/joim.12725 29265670

[fsn34413-bib-0006] Deyama, T. , Nishibe, S. , & Nakazawa, Y. (2001). Constituents and pharmacological effects of Eucommia and Siberian ginseng. Acta Pharmacologica Sinica, 22(12), 1057–1070. 11749801

[fsn34413-bib-0007] Diaz de Barboza, G. , Guizzardi, S. , Moine, L. , & Tolosa de Talamoni, N. (2017). Oxidative stress, antioxidants and intestinal calcium absorption. World Journal of Gastroenterology, 23(16), 2841–2853. 10.3748/wjg.v23.i16.2841 28522903 PMC5413780

[fsn34413-bib-0008] Do, M. , Hur, J. , Choi, J. , Kim, M. , Kim, M. , Kim, Y. , & Ha, S. (2018). *Eucommia ulmoides* ameliorates glucotoxicity by suppressing advanced glycation end‐products in diabetic mice kidney. Nutrients, 10(3), 265. 10.3390/nu10030265 29495397 PMC5872683

[fsn34413-bib-0009] Duan, S. , Lu, F. , Song, D. , Zhang, C. , Zhang, B. , Xing, C. , & Yuan, Y. (2021). Current challenges and future perspectives of renal tubular dysfunction in diabetic kidney disease. Frontiers in Endocrinology, 12, 661185. 10.3389/fendo.2021.661185 34177803 PMC8223745

[fsn34413-bib-0010] Fatchiyah, F. , Setiawan, B. , Sasase, T. , & Ohta, T. (2021). The amelioration of T2DM rat femoral bone achieved by anti‐osteoporosis of caprine CSN1S2 protein through bone morphogenetic protein signaling pathway. Acta Biochimica Polonica, 68(2), 265–275. 10.18388/abp.2020_5553 33964862

[fsn34413-bib-0013] Huang, L. , Lyu, Q. , Zheng, W. , Yang, Q. , & Cao, G. (2021). Traditional application and modern pharmacological research of *Eucommia ulmoides* Oliv. Chinese Medicine, 16(1), 73. 10.1186/s13020-021-00482-7 34362420 PMC8349065

[fsn34413-bib-0014] Hussain, T. , Tan, B. , Liu, G. , Oladele, O. A. , Rahu, N. , Tossou, M. C. , & Yin, Y. (2016). Health‐promoting properties of *Eucommia ulmoides*: A review. Evidence‐based Complementary and Alternative Medicine, 2016, 1–9. 10.1155/2016/5202908 PMC4793136 27042191

[fsn34413-bib-0015] Jiao, H. , Xiao, E. , & Graves, D. T. (2015). Diabetes and its effect on bone and fracture healing. Current Osteoporosis Reports, 13(5), 327–335. 10.1007/s11914-015-0286-8 26254939 PMC4692363

[fsn34413-bib-0016] Kasozi, K. I. , Namubiru, S. , Safiriyu, A. A. , Ninsiima, H. I. , Nakimbugwe, D. , Namayanja, M. , & Valladares, M. B. (2018). Grain Amaranth is associated with improved hepatic and renal calcium metabolism in type 2 diabetes mellitus of male Wistar rats. Evidence‐based Complementary and Alternative Medicine, 2018, 1–10. 10.1155/2018/4098942 PMC6211157 30420893

[fsn34413-bib-0017] Khosla, S. , Samakkarnthai, P. , Monroe, D. G. , & Farr, J. N. (2021). Update on the pathogenesis and treatment of skeletal fragility in type 2 diabetes mellitus. Nature Reviews Endocrinology, 17(11), 685–697. 10.1038/s41574-021-00555-5 PMC8605611 34518671

[fsn34413-bib-0019] Lee, E. , Na, W. , Kang, M. , Kim, Y. , Kim, D. , Oh, H. , Kim, S. , Oh, S. , Park, S. , Park, K. , & Kang, Y. (2021). Hydroxycoumarin Scopoletin inhibits bone loss through enhancing induction of bone turnover markers in a mouse model of type 2 diabetes. Biomedicine, 9(6), 648. 10.3390/biomedicines9060648 PMC8227109 34200167

[fsn34413-bib-0020] Lee, S. , & Kim, I. (2018). Difructose dianhydride improves intestinal calcium absorption, wound healing, and barrier function. Scientific Reports, 8(1), 7813. 10.1038/s41598-018-26295-7 29777169 PMC5959885

[fsn34413-bib-0021] Liu, C. , Zhu, R. , Liu, H. , Li, L. , Chen, B. , Jia, Q. , Wang, L. , Ma, R. , Tian, S. , Wang, M. , Fu, M. , Niu, J. , Orekhov, A. N. , Gao, S. , Zhang, D. , & Zhao, B. (2018). Aqueous extract of Mori folium exerts bone protective effect through regulation of calcium and redox homeostasis via PTH/VDR/CaBP and AGEs/RAGE/Nox4/NF‐κB signaling in diabetic rats. Frontiers in Pharmacology, 9, 1239. 10.3389/fphar.2018.01239 30459613 PMC6233025

[fsn34413-bib-0057] Lu, C. W. , Lee, C. J. , Hsieh, Y. J. , & Hsu, B. G. (2023). Empagliflozin attenuates vascular calcification in mice with chronic kidney disease by regulating the NFR2/HO‐1 anti‐inflammatory pathway through AMPK activation. International Journal of Molecular Sciences, 24(12), 10016. 10.3390/ijms241210016 37373164 PMC10298656

[fsn34413-bib-0022] Luo, B. , Zhou, X. , Tang, Q. , Yin, Y. , Feng, G. , Li, S. , & Chen, L. (2021). Circadian rhythms affect bone reconstruction by regulating bone energy metabolism. Journal of Translational Medicine, 19(1), 410. 10.1186/s12967-021-03068-x 34579752 PMC8477514

[fsn34413-bib-0023] Ma, H. , Wang, X. , Zhang, W. , Li, H. , Zhao, W. , Sun, J. , & Yang, M. (2020). Melatonin suppresses ferroptosis induced by high glucose via activation of the Nrf2/HO‐1 signaling pathway in type 2 diabetic osteoporosis. Oxidative Medicine and Cellular Longevity, 2020, 1–18. 10.1155/2020/9067610 PMC7732386 33343809

[fsn34413-bib-0024] Ma, R. , Zhu, R. , Wang, L. , Guo, Y. , Liu, C. , Liu, H. , Liu, F. , Li, H. , Li, Y. , Fu, M. , & Zhang, D. (2016). Diabetic osteoporosis: A review of its traditional Chinese medicinal use and clinical and preclinical research. Evidence‐based Complementary and Alternative Medicine, 2016, 1–13. 10.1155/2016/3218313 PMC5028800 27698674

[fsn34413-bib-0025] Matikainen, N. , Pekkarinen, T. , Ryhänen, E. M. , & Schalin‐Jäntti, C. (2021). Physiology of calcium homeostasis. Endocrinology and Metabolism Clinics of North America, 50(4), 575–590. 10.1016/j.ecl.2021.07.005 34774235

[fsn34413-bib-0026] Moe, S. (2016). Calcium homeostasis in health and in kidney disease. Comprehensive Physiology, 6(4), 1781–1800. 10.1002/cphy.c150052 27783859

[fsn34413-bib-0027] Naidoo, K. , Ngubane, P. , & Khathi, A. (2022). Investigating the effects of diet‐induced pre‐diabetes on the functioning of calcium‐regulating organs in male Sprague Dawley rats: Effects on selected markers. Frontiers in Endocrinology, 13, 914189. 10.3389/fendo.2022.914189 35898447 PMC9309376

[fsn34413-bib-0028] Nie, X. , Jin, H. , Wen, G. , Xu, J. , An, J. , Liu, X. , Xie, R. , & Tuo, B. (2020). Estrogen regulates duodenal calcium absorption through differential role of estrogen receptor on calcium transport proteins. Digestive Diseases and Sciences, 65(12), 3502–3513. 10.1007/s10620-020-06076-x 31974908

[fsn34413-bib-0051] Pan, Y. , Niu, Y. , Li, C. , Zhai, Y. , Zhang, R. , Guo, X. , & Mei, Q. (2014). Du‐zhong (*Eucommia ulmoides*) prevents disuse‐induced osteoporosis in hind limb suspension rats. The American Journal of Chinese Medicine, 42(1), 143–155. 10.1142/S0192415X14500104 24467541

[fsn34413-bib-0052] Qi, S. , Zheng, H. , Chen, C. , & Jiang, H. (2019). Du‐Zhong (*Eucommia ulmoides* Oliv.) cortex extract alleviates lead acetate‐induced bone loss in rats. Biological Trace Element Research, 187(1), 172–180. 10.1007/s12011-018-1362-6 29740803

[fsn34413-bib-0029] Rathinavelu, S. , Guidry‐Elizondo, C. , & Banu, J. (2018). Molecular modulation of osteoblasts and osteoclasts in type 2 diabetes. Journal of Diabetes Research, 2018, 1–11. 10.1155/2018/6354787 PMC6247387 30525054

[fsn34413-bib-0030] Rivoira, M. , Rodríguez, V. , López, M. P. , & Tolosa de Talamoni, N. (2015). Time dependent changes in the intestinal Ca2+ absorption in rats with type I diabetes mellitus are associated with alterations in the intestinal redox state. Biochimica et Biophysica Acta (BBA) ‐ Molecular Basis of Disease, 1852(3), 386–394. 10.1016/j.bbadis.2014.11.018 25459228

[fsn34413-bib-0031] Saha, S. , Buttari, B. , Panieri, E. , Profumo, E. , & Saso, L. (2020). An overview of *Nrf2* signaling pathway and its role in inflammation. Molecules, 25(22), 5474. 10.3390/molecules25225474 33238435 PMC7700122

[fsn34413-bib-0054] Savic, N. , Markelic, M. , Stancic, A. , Velickovic, K. , Grigorov, I. , Vucetic, M. , Martinovic, V. , Gudelj, A. , & Otasevic, V. (2024). Sulforaphane prevents diabetes‐induced hepatic ferroptosis by activating Nrf2 signaling axis. BioFactors (Oxford, England), 50(4), 810–827. 10.1002/biof.2042 38299761

[fsn34413-bib-0032] Shanbhogue, V. V. , Mitchell, D. M. , Rosen, C. J. , & Bouxsein, M. L. (2016). Type 2 diabetes and the skeleton: New insights into sweet bones. The Lancet Diabetes & Endocrinology, 4(2), 159–173. 10.1016/S2213-8587(15)00283-1 26365605

[fsn34413-bib-0033] Sumida, Y. , Shima, T. , Mitsumoto, Y. , Katayama, T. , Umemura, A. , Yamaguchi, K. , Itoh, Y. , Yoneda, M. , & Okanoue, T. (2020). Epidemiology, pathogenesis, and diagnostic strategy of diabetic liver disease in Japan. International Journal of Molecular Sciences, 21(12), 4337. 10.3390/ijms21124337 32570776 PMC7352222

[fsn34413-bib-0034] Tanase, D. M. , Gosav, E. M. , Costea, C. F. , Ciocoiu, M. , Lacatusu, C. M. , Maranduca, M. A. , Ouatu, A. , & Floria, M. (2020). The intricate relationship between type 2 diabetes mellitus (T2DM), insulin resistance (IR), and nonalcoholic fatty liver disease (NAFLD). Journal of Diabetes Research, 2020, 1–16. 10.1155/2020/3920196 PMC7424491 32832560

[fsn34413-bib-0035] Tinawi, M. (2021). Disorders of calcium metabolism: Hypocalcemia and hypercalcemia. Cureus, 13(1), e12420. 10.7759/cureus.12420 33542868 PMC7849212

[fsn34413-bib-0038] Weaver, C. M. , & Peacock, M. (2011). Calcium. Advances in Nutrition, 2(3), 290–292. 10.3945/an.111.000463 22332061 PMC3090164

[fsn34413-bib-0050] Weaver, C. M. , & Peacock, M. (2019). Calcium. Advances in Nutrition, 10(3), 546–548. 10.1093/advances/nmy086 30915443 PMC6520034

[fsn34413-bib-0039] Wongdee, K. , Krishnamra, N. , & Charoenphandhu, N. (2017). Derangement of calcium metabolism in diabetes mellitus: Negative outcome from the synergy between impaired bone turnover and intestinal calcium absorption. The Journal of Physiological Sciences, 67(1), 71–81. 10.1007/s12576-016-0487-7 27671701 PMC10717635

[fsn34413-bib-0053] Wu, J. , Zhang, Z. , Wu, Q. , Zhang, L. , Chen, Z. , Zhao, H. , Wu, X. , Zhao, Y. , Zhang, C. , Ge, J. , & Liu, H. (2023). Antioxidative effect of Periplaneta Americana extract on dextran sulfate sodium‐induced ulcerative colitis through activation of the Nrf2 signal. Pharmaceutical Biology, 61(1), 949–962. 10.1080/13880209.2023.2220351 37334466 PMC10599263

[fsn34413-bib-0040] Xiao, D. , Yuan, D. , Tan, B. , Wang, J. , Liu, Y. , & Tan, B. (2019). The role of *Nrf2* signaling pathway in *Eucommia ulmoides* flavones regulating oxidative stress in the intestine of piglets. Oxidative Medicine and Cellular Longevity, 2019, 1–9. 10.1155/2019/9719618 PMC6745127 31565157

[fsn34413-bib-0041] Xiao, J. , Zhang, G. , Chen, B. , He, Q. , Mai, J. , Chen, W. , Pan, Z. , Yang, J. , Li, J. , Ma, Y. , Wang, T. , & Wang, H. (2023). Quercetin protects against iron overload‐induced osteoporosis through activating the Nrf2/HO‐1 pathway. Life Sciences, 322, 121326. 10.1016/j.lfs.2022.121326 36639053

[fsn34413-bib-0058] Xie, G. P. , Jiang, N. , Wang, S. N. , Qi, R. Z. , Wang, L. , Zhao, P. R. , Liang, L. , & Yu, B. (2015). *Eucommia ulmoides* Oliv. bark aqueous extract inhibits osteoarthritis in a rat model of osteoarthritis. Journal of Ethnopharmacology, 162, 148–154. 10.1016/j.jep.2014.12.061 25575468

[fsn34413-bib-0055] Xu, C. , Xia, L. , Xu, D. , Liu, Y. , Jin, P. , Zhai, M. , Mao, Y. , Wang, Y. , Wen, A. , Yang, J. , & Yang, L. (2024). Cardioprotective effects of asiaticoside against diabetic cardiomyopathy: Activation of the AMPK/Nrf2 pathway. Journal of Cellular and Molecular Medicine, 28(2), e18055. 10.1111/jcmm.18055 38113341 PMC10826442

[fsn34413-bib-0042] Yu, S. , Zhang, C. , Xu, X. , Sun, J. , Zhang, L. , & Yu, P. (2015). Ursolic acid derivative ameliorates streptozotocin‐induced diabestic bone deleterious effects in mice. International Journal of Clinical and Experimental Pathology, 8(4), 3681–3690. 26097549 PMC4466936

[fsn34413-bib-0043] Zhang, X. , Yu, Y. , Lei, H. , Cai, Y. , Shen, J. , Zhu, P. , He, Q. , & Zhao, M. (2020). The Nrf‐2/HO‐1 signaling Axis: A ray of Hope in cardiovascular diseases. Cardiology Research and Practice, 2020, 1–9. 10.1155/2020/5695723 PMC7204387 32411446

[fsn34413-bib-0044] Zhang, Y. , Papasian, C. J. , & Deng, H.‐W. (2011). Alteration of vitamin D metabolic enzyme expression and calcium transporter abundance in kidney involved in type 1 diabetes‐induced bone loss. Osteoporosis International, 22(6), 1781–1788. 10.1007/s00198-010-1404-1 20878391 PMC4537183

[fsn34413-bib-0046] Zhao, W. , Zhang, W. , Ma, H. , & Yang, M. (2020). NIPA2 regulates osteoblast function by modulating mitophagy in type 2 diabetes osteoporosis. Scientific Reports, 10(1), 3078. 10.1038/s41598-020-59743-4 32080264 PMC7033235

[fsn34413-bib-0047] Zhao, Y. , Qiu, J. , Chen, T. , Wang, S. , Liu, S. , Huang, H. , & Wan, L. (2021). The efficacy and safety of traditional Chinese medicine's tonifying‐kidney, strengthening‐spleen, and invigorating‐blood circulation (Bushen‐Jianpi‐Huoxue) principle for type 2 diabetes mellitus with osteoporosis. Medicine, 100(12), e25197. 10.1097/MD.0000000000025197 33761702 PMC9282049

[fsn34413-bib-0048] Zhong, H. , Yuan, Y. , Xie, W. , Chen, M. , & He, X. (2016). Type 2 diabetes mellitus is associated with more serious small intestinal mucosal injuries. PLoS One, 11(9), e0162354. 10.1371/journal.pone.0162354 27598308 PMC5012602

[fsn34413-bib-0049] Zhu, C. , Shen, S. , Zhang, S. , Huang, M. , Zhang, L. , & Chen, X. (2022). Autophagy in bone remodeling: A regulator of oxidative stress. Frontiers in Endocrinology, 13, 898634. 10.3389/fendo.2022.898634 35846332 PMC9279723

